# 4-Chloro-*N*-[3-methyl-1-(5-thioxo-4,5-dihydro-1,3,4-oxadiazol-2-yl)but­yl]benzamide

**DOI:** 10.1107/S1600536810017368

**Published:** 2010-05-19

**Authors:** Yu-Gang Yan, Guo-Gang Tu, Ling-Dong Wang, Jian Liu, Shao-Hua Li

**Affiliations:** a Department of Medicinal Chemistry, NanChang University School of Pharmaceutical Science, 330006 NanChang, JiangXi, People’s Republic of China

## Abstract

In the title compound, C_14_H_16_ClN_3_O_2_S, the dihedral angle between the 4-chloro­phenyl and 1,3,4-oxadiazole rings is 67.1 (1)° and the orientation of the amide N—H and C=O bonds is *anti*. In the crystal, mol­ecules are linked by N—H⋯O and N—H⋯S hydrogen bonds.

## Related literature

For the biological properties of thia­diazo­les, see: Tu *et al.* (2008 ▶). For details of the synthesis, see: Ginzel *et al.* (1989 ▶); Boland *et al.* (2006 ▶); Havaldar & Patil (2009 ▶); Shriner & Furrow (1955 ▶). For related structures, see: Du *et al.* (2004 ▶); Ziyaev *et al.* (1992 ▶); Zareef *et al.* (2006 ▶).
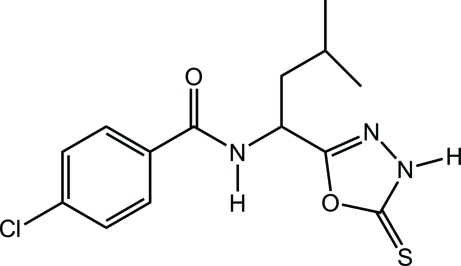


## Experimental

### 

#### Crystal data

C_14_H_16_ClN_3_O_2_S
*M* *_r_* = 325.81
Orthorhombic, 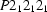

*a* = 6.0171 (6) Å
*b* = 15.3120 (15) Å
*c* = 18.1493 (17) Å
*V* = 1672.2 (3) Å^3^
*Z* = 4
Mo *K*α radiation
μ = 0.36 mm^−1^
*T* = 298 K
0.42 × 0.22 × 0.18 mm

#### Data collection

Bruker SMART CCD diffractometer
Absorption correction: multi-scan (*SADABS*; Sheldrick, 1996 ▶) *T*_min_ = 0.864, *T*_max_ = 0.938
7892 measured reflections
2951 independent reflections
1447 reflections with *I* > 2σ(*I*)
*R*_int_ = 0.056

#### Refinement

*R*[*F* ^2^ > 2σ(*F* ^2^)] = 0.050
*wR*(*F* ^2^) = 0.108
*S* = 1.10
2951 reflections
193 parameters
H-atom parameters constrained
Δρ_max_ = 0.31 e Å^−3^
Δρ_min_ = −0.32 e Å^−3^
Absolute structure: Flack (1983 ▶), 1219 Friedel pairs
Flack parameter: −0.09 (14)

### 

Data collection: *SMART* (Bruker, 2001 ▶); cell refinement: *SAINT* (Bruker, 2001 ▶); data reduction: *SAINT*; program(s) used to solve structure: *SHELXS97* (Sheldrick, 2008 ▶); program(s) used to refine structure: *SHELXL97* (Sheldrick, 2008 ▶); molecular graphics: *SHELXTL* (Sheldrick, 2008 ▶); software used to prepare material for publication: *SHELXTL* and *publCIF* (Westrip, 2010 ▶).

## Supplementary Material

Crystal structure: contains datablocks I, global. DOI: 10.1107/S1600536810017368/hb5439sup1.cif

Structure factors: contains datablocks I. DOI: 10.1107/S1600536810017368/hb5439Isup2.hkl

Additional supplementary materials:  crystallographic information; 3D view; checkCIF report

## Figures and Tables

**Table 1:** Hydrogen-bond geometry (Å, °)

*D*—H⋯*A*	*D*—H	H⋯*A*	*D*⋯*A*	*D*—H⋯*A*
N1—H1⋯O2^i^	0.86	1.87	2.720 (6)	171
N3—H3⋯S1^ii^	0.86	2.78	3.495 (4)	142

Symmetry codes: (i) 
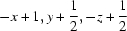
; (ii) 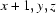.

**Fig. 1.**
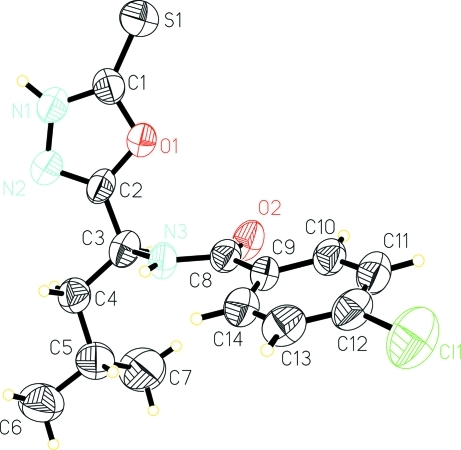
Molecular structure of (I) with displacement ellipsoids drawn at the 50% probability level.

**Fig. 2.**
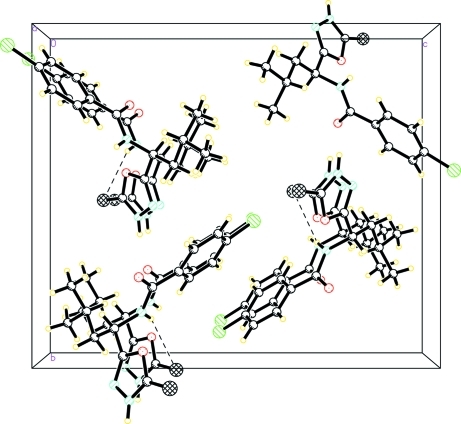
The crystal packing of (I), viewed along the a axis with hydrogen bonds drawn as dashed lines.

## supplementary crystallographic information

### Comment

The present oxadiazole derivate is in continuation to our previous work of the thiadiazole scaffold compounds and their biological activity (Tu *et al.*, 2008). The title compound (Figure 1) was synthesized according to literature procedures (Ginzel *et al.*,  1989; Boland *et al.*, 2006; Havaldar & Patil 2009). Here, we report the structure of the title compound.

The oxadiazole ring is essentially planar and is inclined at 67.1 (1)° with respect to the *p*-cholobenzene ring. The N2=C2 and S1=C1 double bonds agree with the corresponding distances in three structures containing similar systems (Du *et al.*,  2004; Ziyaev *et al.*, 1992; Zareef *et al.*, 2006). The conformations of the N—H and C=O bonds are *anti* with respect to each other. The structure is stabilized by a network of intermolecular hydrogen bonds of the type N—H···S (Table 1, Figure 2).

### Experimental

To a stirred solution of DL-leucine methyl ester hydrochloride (0.03 mol) in CH_2_Cl_2_ (20 ml) was added triethylamine (0.06 mol) at 273 K. After 0.5 h, a solution of *p*-chlorobenzoic acid chloride (0.03 mol) in CH_2_Cl_2_ (10 ml) was added. The mixture was stirred for 2 h at 273 K, then allowed to warm to r.t. for 24 h. Washed with 10% HCl, 1 N NaOH and water. The organic layer was evaporated *in vacuo* and the residue was recrystallized from methanol to give corresponding amides as a white solid.

A mixture of the amides (0.02 mol) and 80% hydrazine monohydrate (0.04 mol) in absolute methanol (20 ml) was heated under reflux over night. After cooling, a white solid was separated and recrystallized from methanol to give corresponding hydrazide.

A mixture of the hydrazide (0.01 mol), KOH (0.01 mol), CS_2_ (0.05 mol), and ethanol (70 ml) was heated under reflux with stirring for 12 h. Ethanol was distilled off under reduced pressure and the residue was dissolved in water and then acidified with 10% HCl. The resulting precipitate was filtered, washed with water, and recrystallized from ethanol. Colourless blocks of (I) precipitated after several days.

### Refinement

H atoms were positioned geometrically and refined using a riding model using SHELXL97 default values (Uiso(H) = 1.2 Ueq(C) for CH and CH_2_ groups and Uiso(H) = 1.5 Ueq(C) for CH_3_).

### Crystal data

**Table:**

C_14_H_16_ClN_3_O_2_S	*F*(000) = 680
*M**_r_* = 325.81	*D*_x_ = 1.294 Mg m^−^^3^
Orthorhombic, *P*2_1_2_1_2_1_	Mo *K*α radiation, λ = 0.71073 Å
Hall symbol: P 2ac 2ab	Cell parameters from 2117 reflections
*a* = 6.0171 (6) Å	θ = 2.6–21.7°
*b* = 15.3120 (15) Å	µ = 0.36 mm^−^^1^
*c* = 18.1493 (17) Å	*T* = 298 K
*V* = 1672.2 (3) Å^3^	Block, colourless
*Z* = 4	0.42 × 0.22 × 0.18 mm

### Data collection

**Table:**

Bruker SMART CCD diffractometer	2951 independent reflections
Radiation source: fine-focus sealed tube	1447 reflections with *I* > 2σ(*I*)
graphite	*R*_int_ = 0.056
ω scans	θ_max_ = 25.0°, θ_min_ = 1.7°
Absorption correction: multi-scan (*SADABS*; Sheldrick, 1996)	*h* = −7→7
*T*_min_ = 0.864, *T*_max_ = 0.938	*k* = −18→11
7892 measured reflections	*l* = −21→16

### Refinement

**Table:**

Refinement on *F*^2^	Hydrogen site location: inferred from neighbouring sites
Least-squares matrix: full	H-atom parameters constrained
*R*[*F*^2^ > 2σ(*F*^2^)] = 0.050	*w* = 1/[σ^2^(*F*_o_^2^) + (0.0147*P*)^2^ + 1.0529*P*] where *P* = (*F*_o_^2^ + 2*F*_c_^2^)/3
*wR*(*F*^2^) = 0.108	(Δ/σ)_max_ < 0.001
*S* = 1.10	Δρ_max_ = 0.31 e Å^−^^3^
2951 reflections	Δρ_min_ = −0.32 e Å^−^^3^
193 parameters	Extinction correction: *SHELXL97* (Sheldrick, 2008), Fc^*^=kFc[1+0.001xFc^2^λ^3^/sin(2θ)]^-1/4^
0 restraints	Extinction coefficient: 0.0034 (7)
Primary atom site location: structure-invariant direct methods	Absolute structure: Flack (1983), 1219 Friedel pairs
Secondary atom site location: difference Fourier map	Flack parameter: −0.09 (14)

### Special details

**Table:**

Geometry. All esds (except the esd in the dihedral angle between two l.s. planes) are estimated using the full covariance matrix. The cell esds are taken into account individually in the estimation of esds in distances, angles and torsion angles; correlations between esds in cell parameters are only used when they are defined by crystal symmetry. An approximate (isotropic) treatment of cell esds is used for estimating esds involving l.s. planes.
Refinement. Refinement of *F*^2^ against ALL reflections. The weighted *R*-factor wR and goodness of fit *S* are based on *F*^2^, conventional *R*-factors *R* are based on *F*, with *F* set to zero for negative *F*^2^. The threshold expression of *F*^2^ > σ(*F*^2^) is used only for calculating *R*-factors(gt) etc. and is not relevant to the choice of reflections for refinement. *R*-factors based on *F*^2^ are statistically about twice as large as those based on *F*, and *R*- factors based on ALL data will be even larger.

### Fractional atomic coordinates and isotropic or equivalent isotropic displacement parameters (Å^2^)

**Table:**

	*x*	*y*	*z*	*U*_iso_*/*U*_eq_	
Cl1	1.2875 (3)	0.07537 (10)	−0.04447 (8)	0.1082 (7)	
N1	0.4961 (8)	0.5638 (3)	0.2486 (2)	0.0653 (12)	
H1	0.4553	0.6175	0.2515	0.078*	
N2	0.6705 (8)	0.5299 (2)	0.2891 (2)	0.0679 (12)	
N3	0.9177 (6)	0.3351 (2)	0.22952 (19)	0.0530 (10)	
H3	1.0323	0.3577	0.2086	0.064*	
O1	0.5141 (6)	0.4303 (2)	0.21792 (16)	0.0650 (10)	
O2	0.6632 (6)	0.22820 (19)	0.23058 (17)	0.0672 (10)	
S1	0.1860 (3)	0.51382 (10)	0.14928 (8)	0.0902 (5)	
C1	0.3976 (8)	0.5066 (3)	0.2049 (2)	0.0601 (13)	
C2	0.6745 (10)	0.4498 (3)	0.2683 (2)	0.0555 (13)	
C3	0.8216 (9)	0.3780 (3)	0.2935 (2)	0.0572 (13)	
H3A	0.7295	0.3352	0.3195	0.069*	
C4	0.9999 (9)	0.4102 (3)	0.3469 (2)	0.0628 (14)	
H4A	1.0827	0.4567	0.3233	0.075*	
H4B	0.9271	0.4348	0.3898	0.075*	
C5	1.1631 (10)	0.3406 (3)	0.3725 (3)	0.0793 (17)	
H5	1.2452	0.3206	0.3290	0.095*	
C6	1.3329 (10)	0.3803 (4)	0.4260 (3)	0.097 (2)	
H6A	1.2604	0.3948	0.4715	0.145*	
H6B	1.3955	0.4321	0.4046	0.145*	
H6C	1.4490	0.3387	0.4353	0.145*	
C7	1.0511 (12)	0.2623 (4)	0.4053 (3)	0.129 (3)	
H7A	0.9645	0.2799	0.4471	0.193*	
H7B	1.1615	0.2208	0.4205	0.193*	
H7C	0.9557	0.2360	0.3692	0.193*	
C8	0.8305 (9)	0.2601 (3)	0.2021 (2)	0.0520 (12)	
C9	0.9491 (9)	0.2185 (3)	0.1398 (3)	0.0518 (13)	
C10	0.8393 (9)	0.1520 (3)	0.1025 (2)	0.0575 (13)	
H10	0.6966	0.1359	0.1168	0.069*	
C11	0.9413 (10)	0.1093 (3)	0.0440 (3)	0.0673 (15)	
H11	0.8661	0.0660	0.0181	0.081*	
C12	1.1541 (11)	0.1319 (3)	0.0247 (3)	0.0663 (15)	
C13	1.2613 (9)	0.1986 (3)	0.0598 (3)	0.0681 (15)	
H13	1.4027	0.2153	0.0446	0.082*	
C14	1.1604 (9)	0.2411 (3)	0.1174 (3)	0.0639 (14)	
H14	1.2355	0.2858	0.1416	0.077*	

### Atomic displacement parameters (Å^2^)

**Table:**

	*U*^11^	*U*^22^	*U*^33^	*U*^12^	*U*^13^	*U*^23^
Cl1	0.1383 (16)	0.0987 (12)	0.0876 (10)	0.0156 (12)	0.0383 (11)	−0.0126 (9)
N1	0.069 (3)	0.046 (3)	0.081 (3)	0.002 (3)	−0.002 (3)	−0.005 (2)
N2	0.078 (3)	0.047 (3)	0.079 (3)	0.005 (3)	−0.012 (3)	−0.004 (2)
N3	0.050 (3)	0.044 (2)	0.066 (3)	−0.006 (2)	0.007 (2)	−0.0108 (19)
O1	0.079 (3)	0.045 (2)	0.071 (2)	0.002 (2)	−0.019 (2)	−0.0068 (17)
O2	0.069 (3)	0.046 (2)	0.086 (2)	−0.004 (2)	0.016 (2)	−0.0039 (17)
S1	0.0914 (12)	0.0756 (10)	0.1037 (11)	−0.0050 (10)	−0.0315 (10)	0.0091 (9)
C1	0.064 (4)	0.058 (3)	0.058 (3)	−0.009 (3)	−0.004 (3)	−0.002 (3)
C2	0.074 (4)	0.037 (3)	0.056 (3)	0.004 (3)	0.001 (3)	−0.005 (2)
C3	0.072 (4)	0.046 (3)	0.053 (3)	0.001 (3)	0.009 (3)	−0.002 (2)
C4	0.074 (4)	0.056 (3)	0.058 (3)	0.001 (3)	−0.007 (3)	−0.004 (3)
C5	0.088 (5)	0.075 (4)	0.074 (4)	0.001 (4)	−0.015 (4)	0.007 (3)
C6	0.094 (5)	0.109 (5)	0.088 (4)	0.010 (4)	−0.017 (4)	0.003 (3)
C7	0.140 (7)	0.092 (5)	0.154 (6)	−0.018 (5)	−0.040 (5)	0.056 (5)
C8	0.056 (3)	0.043 (3)	0.057 (3)	0.000 (3)	0.000 (3)	0.001 (2)
C9	0.057 (3)	0.037 (3)	0.062 (3)	−0.002 (3)	−0.006 (3)	−0.003 (2)
C10	0.062 (4)	0.044 (3)	0.067 (3)	−0.004 (3)	0.001 (3)	0.002 (2)
C11	0.097 (5)	0.048 (3)	0.056 (3)	0.001 (3)	−0.001 (3)	−0.001 (3)
C12	0.084 (5)	0.055 (3)	0.059 (3)	0.011 (4)	0.007 (3)	0.005 (3)
C13	0.063 (4)	0.068 (4)	0.074 (3)	0.005 (3)	0.014 (3)	0.006 (3)
C14	0.067 (4)	0.051 (3)	0.074 (3)	−0.003 (3)	−0.002 (3)	0.000 (3)

### Geometric parameters (Å, °)

**Table:**

Cl1—C12	1.723 (5)	C5—C6	1.535 (7)
N1—C1	1.321 (5)	C5—H5	0.9800
N1—N2	1.382 (5)	C6—H6A	0.9600
N1—H1	0.8600	C6—H6B	0.9600
N2—C2	1.283 (5)	C6—H6C	0.9600
N3—C8	1.357 (5)	C7—H7A	0.9600
N3—C3	1.454 (5)	C7—H7B	0.9600
N3—H3	0.8600	C7—H7C	0.9600
O1—C2	1.363 (5)	C8—C9	1.481 (6)
O1—C1	1.382 (5)	C9—C14	1.379 (6)
O2—C8	1.232 (5)	C9—C10	1.390 (6)
S1—C1	1.629 (5)	C10—C11	1.390 (6)
C2—C3	1.483 (6)	C10—H10	0.9300
C3—C4	1.527 (6)	C11—C12	1.372 (7)
C3—H3A	0.9800	C11—H11	0.9300
C4—C5	1.522 (6)	C12—C13	1.366 (6)
C4—H4A	0.9700	C13—C14	1.373 (6)
C4—H4B	0.9700	C13—H13	0.9300
C5—C7	1.498 (7)	C14—H14	0.9300
			
C1—N1—N2	114.3 (4)	C5—C6—H6B	109.5
C1—N1—H1	122.9	H6A—C6—H6B	109.5
N2—N1—H1	122.9	C5—C6—H6C	109.5
C2—N2—N1	102.5 (4)	H6A—C6—H6C	109.5
C8—N3—C3	121.5 (4)	H6B—C6—H6C	109.5
C8—N3—H3	119.3	C5—C7—H7A	109.5
C3—N3—H3	119.3	C5—C7—H7B	109.5
C2—O1—C1	106.8 (3)	H7A—C7—H7B	109.5
N1—C1—O1	103.3 (4)	C5—C7—H7C	109.5
N1—C1—S1	132.6 (4)	H7A—C7—H7C	109.5
O1—C1—S1	124.0 (4)	H7B—C7—H7C	109.5
N2—C2—O1	113.1 (5)	O2—C8—N3	119.8 (4)
N2—C2—C3	128.9 (5)	O2—C8—C9	122.9 (4)
O1—C2—C3	117.9 (4)	N3—C8—C9	117.2 (4)
N3—C3—C2	109.0 (3)	C14—C9—C10	118.6 (5)
N3—C3—C4	111.9 (4)	C14—C9—C8	124.2 (4)
C2—C3—C4	112.1 (4)	C10—C9—C8	117.3 (5)
N3—C3—H3A	107.9	C11—C10—C9	120.4 (5)
C2—C3—H3A	107.9	C11—C10—H10	119.8
C4—C3—H3A	107.9	C9—C10—H10	119.8
C5—C4—C3	114.9 (4)	C12—C11—C10	119.3 (5)
C5—C4—H4A	108.5	C12—C11—H11	120.4
C3—C4—H4A	108.5	C10—C11—H11	120.4
C5—C4—H4B	108.5	C13—C12—C11	120.7 (5)
C3—C4—H4B	108.5	C13—C12—Cl1	119.7 (5)
H4A—C4—H4B	107.5	C11—C12—Cl1	119.6 (5)
C7—C5—C4	113.0 (5)	C12—C13—C14	120.1 (5)
C7—C5—C6	111.4 (5)	C12—C13—H13	120.0
C4—C5—C6	110.2 (4)	C14—C13—H13	120.0
C7—C5—H5	107.3	C13—C14—C9	120.9 (5)
C4—C5—H5	107.3	C13—C14—H14	119.6
C6—C5—H5	107.3	C9—C14—H14	119.6
C5—C6—H6A	109.5		
			
C1—N1—N2—C2	0.1 (6)	C3—C4—C5—C6	−179.7 (4)
N2—N1—C1—O1	0.3 (5)	C3—N3—C8—O2	1.7 (7)
N2—N1—C1—S1	178.2 (4)	C3—N3—C8—C9	−176.2 (4)
C2—O1—C1—N1	−0.5 (5)	O2—C8—C9—C14	−165.1 (4)
C2—O1—C1—S1	−178.7 (3)	N3—C8—C9—C14	12.7 (7)
N1—N2—C2—O1	−0.5 (5)	O2—C8—C9—C10	14.3 (7)
N1—N2—C2—C3	−178.1 (5)	N3—C8—C9—C10	−167.9 (4)
C1—O1—C2—N2	0.7 (5)	C14—C9—C10—C11	0.1 (7)
C1—O1—C2—C3	178.6 (4)	C8—C9—C10—C11	−179.3 (4)
C8—N3—C3—C2	−98.1 (5)	C9—C10—C11—C12	1.8 (7)
C8—N3—C3—C4	137.4 (4)	C10—C11—C12—C13	−3.5 (7)
N2—C2—C3—N3	−129.7 (5)	C10—C11—C12—Cl1	176.5 (3)
O1—C2—C3—N3	52.8 (6)	C11—C12—C13—C14	3.2 (8)
N2—C2—C3—C4	−5.2 (8)	Cl1—C12—C13—C14	−176.7 (4)
O1—C2—C3—C4	177.2 (4)	C12—C13—C14—C9	−1.2 (7)
N3—C3—C4—C5	−54.7 (6)	C10—C9—C14—C13	−0.4 (7)
C2—C3—C4—C5	−177.5 (4)	C8—C9—C14—C13	178.9 (4)
C3—C4—C5—C7	−54.3 (6)		

### Hydrogen-bond geometry (Å, °)

**Table:**

*D*—H···*A*	*D*—H	H···*A*	*D*···*A*	*D*—H···*A*
N1—H1···O2^i^	0.86	1.87	2.720 (6)	171
N3—H3···S1^ii^	0.86	2.78	3.495 (4)	142

Symmetry codes: (i) −*x*+1, *y*+1/2, −*z*+1/2; (ii) *x*+1, *y*, *z*.
